# Genetic Divergence in the Absence of Strong Ecological Differences Between Coexisting White and Common Atlantic Marine Stickleback

**DOI:** 10.1002/ece3.73655

**Published:** 2026-05-13

**Authors:** Kieran Samuk, Hannah Visty, Dolph Schluter

**Affiliations:** ^1^ Department of Evolution, Ecology, and Organismal Biology The University of California Riverside California USA; ^2^ Zoology Department, and Biodiversity Research Centre University of British Columbia Vancouver British Columbia Canada

## Abstract

Identifying taxa in the earliest phases of speciation is critical for understanding how reproductive isolation arises. In Nova Scotia, Canada, “white” threespine stickleback co‐occur with common marine stickleback but differ in size, nuptial coloration, nesting behavior, and parental care, raising the possibility that they represent a distinct ecotype with some degree of reproductive isolation. We combined population genomics, morphometrics, and stable isotope analysis to test whether white stickleback represent a distinct lineage and whether they have diverged along ecological axes as have freshwater stickleback populations. Genotyping‐by‐sequencing revealed that male and female white stickleback form a genetic cluster distinct from sympatric common stickleback with evidence of ongoing gene flow yet with very low overall genomic divergence (F_ST_ ≈ 0.01). Genetic differences were distributed across many loci rather than localized to a single genomic region. Morphological and isotopic analyses revealed no differences in most classic ecological traits (body shape, armor, gill rakers, or trophic niche). Instead, whites are smaller‐bodied, paler, and exhibit shorter spines, reduced testes size, and smaller but more numerous eggs compared to common stickleback. These results indicate that white stickleback are genetically distinct from the common Atlantic threespine stickleback but have not diverged conspicuously in their ecology other than in size, suggesting that their differentiation is driven by reproductive and sexual traits rather than trophic specialization. The white stickleback thus represent a promising new system for investigating the interplay of sexual selection, reproductive strategy, and gene flow in the early stages of speciation.

## Graphical abstract

We examined whether the “white” threespine stickleback of Nova Scotia represents a nascent species distinct from sympatric common marine sticklebacks. Genomic analyses revealed that whites form a unique genetic cluster with low but widespread divergence across the genome despite evidence of gene flow, while morphometric and isotopic data indicated minimal ecological differentiation. Instead, phenotypic divergence was concentrated in reproductive and sexual traits, suggesting that differences in reproductive strategy, rather than trophic specialization, may underlie reproductive isolation in this system.

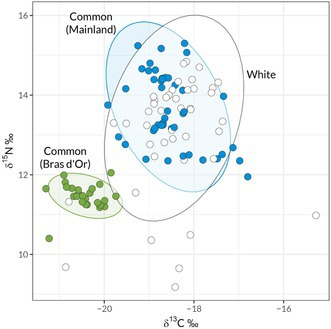

## 1 Introduction

Understanding the genetic and phenotypic changes that lead to the formation of new species remains a major goal of evolutionary biology (Coyne and Orr 2004; Knott et al. 2012). While recent advances in genomics have provided insight into some aspects of the evolution of reproductive isolation (RI), there are still many unanswered questions about how isolation evolves in natural systems (Butlin et al. 2012; Noor and Feder 2006; Wu and Ting 2004). Among these open questions are: How do new species arise in the face of gene flow? What role does divergent natural selection play in the formation of species boundaries? What phenotypic and genetic changes initiate the speciation process? The present study bears most directly on the first and third of these questions.

Choosing a study system to approach these questions is complicated by the fact that species vary in their progress along the “speciation continuum” (Roux et al. 2016). It is generally agreed that younger species are the most useful for studying speciation (Coyne and Orr 2004; Knott et al. 2012; Via 2009). This is because recently diverged species avoid a key problem with the study of the evolution of reproductive isolation (hereafter, RI): as diverging populations proceed toward reproductive isolation, new reproductive barriers arise and mask those that formed at the onset of speciation (Butlin et al. 2012; Coyne and Orr 2004; Roux et al. 2016; Via 2009). These later‐forming barriers may help maintain RI (e.g., by causing postzygotic RI), but they are not necessarily informative of the key barriers that originally caused speciation to occur (Coyne and Orr 2004; Orr 2005; Price 2008). For example, a late‐evolved lethal intrinsic incompatibility between two species could mask the role of poor ecological performance of hybrids because hybrids are never formed (Butlin et al. 2012). Later‐accumulating barriers also attenuate gene flow and cause genome‐wide divergence to increase, reducing the power of divergence‐based methods for detecting loci involved in RI (Egan et al. 2013; Feder et al. 2012; Noor and Feder 2006). Thus, we can maximize our ability to find the genetic and phenotypic changes that initiate speciation by studying recently diverged taxa, i.e., young/incipient species.

The utility of young co‐occurring species for identifying RI has become particularly apparent in the age of genomics (Feder et al. 2012; Ravinet et al. 2017). This is because young species often exhibit incomplete reproductive isolation, which allows gene flow to homogenize parts of the genome that are not involved in the maintenance of species differences, amplifying the genomic signatures of divergence at RI loci (Rogers and Bernatchez 2006; Stephan et al. 2010; Wu 2001). Despite these obvious advantages, there are still only a handful of developed systems for studying the very early stages of speciation, particularly in the presence of gene flow. Some examples of such systems include *Rhagoletus* apple/hawthorn flies, *Littorina* intertidal snails, *Helianthus* dune sunflowers, and *Timema* walking sticks, which have all begun to yield key insights into the speciation process (Feder et al. 2003; Le Moan et al. 2023; Nosil et al. 2005; Ostevik et al. 2016; Rieseberg et al. 2012). A complete picture of the early phases of speciation will require additional study systems, particularly those with developed genomic resources.

The threespine stickleback ( *Gasterosteus aculeatus* ) species complex is thought to harbor many young species. In five postglacial lake systems, stickleback species pairs are genetically diverged and exhibit strong but incomplete RI (Lavin and Mcphail 1993; Gow et al. 2008; Schluter et al. 2025). Gene flow has apparently occurred throughout divergence (Wang 2018), and RI is mediated largely by their ecological differences (e.g., selection against hybrids with mismatched trophic traits; Arnegard et al. 2014, Thompson et al. 2022, Schluter et al. 2025) and by assortative mating on the basis of body size and shape (Conte and Schluter 2013; Rundle et al. 2005; Bay et al. 2017). This pattern of divergence along ecological axes is consistent with the idea that speciation with gene flow can result from strong, divergent natural selection (e.g., provided by different trophic niches). However, many of the best‐studied population and species pairs have moderate to high levels of genome‐wide genetic differentiation (Hohenlohe et al. 2010; Jones et al. 2012; Reid et al. 2021; Roesti et al. 2012). Thus, we have an incomplete sample of divergence/speciation continuum in stickleback and are limited in our ability to probe the genetic and phenotypic changes that underlie the crucial initial stages of speciation. To amend this, new systems are needed in which stickleback species have evolved recently and still exchange genes.

### 1.1 The White Stickleback

The “white” threespine stickleback from Nova Scotia, Canada, may be one such system (Blouw and Hagen 1990). White stickleback appear to be distinct from common marine stickleback (hereafter “common stickleback”), and both types are broadly sympatric in marine and estuarine environments in Nova Scotia (Blouw and Hagen 1990). Male white stickleback build nests near the shore (sometimes in the intertidal), and use filamentous algae rather than sand and gravel as nesting substrate (Jamieson et al. 1992a, 1992b; Macdonald et al. 1995). When on the breeding grounds, male white stickleback exhibit striking pearlescent‐white breeding colors instead of the more common olive/blue colors (Blouw and Hagen 1990). Intriguingly, male white stickleback also lack the classic paternal care behaviors characteristic of male common stickleback: instead of caring for eggs after fertilization, white males carry eggs away from their nest (often out of their territory entirely), disperse them into the surrounding algae, and return to soliciting matings from females (Blouw 1996; Jamieson et al. 1992b). Male white stickleback are also on average ~20% shorter in body length than common male stickleback, resulting in a bimodal distribution of male body sizes at sites where both are found (Blouw and Hagen 1990).

Recent work has explored a wide variety of phenotypic and genetic differences between white and common stickleback. The difference in male nuptial coloration appears to be caused by a reduced density and extent of melanophores in the skin of white males (Haley et al. 2019). Parenting white and common males also differ in total gene expression during the courtship and parental phases (Barbasch et al. 2024), which appears to be underlain by changes in expression in specific regions of the brain (Dan et al. 2024). Differences in parental care behavior have also now been mapped to specific QTL (Behrens, Maciejewski, et al. 2025; Behrens, Tucker, et al. 2025), implying a genetic basis. Finally, lab‐reared F1 and F2 hybrids between white and common stickleback also exhibit transgressive and putatively maladaptive parental care behavior, including reduced fanning behavior, a mix of common and white parental care strategies (e.g., nest building with no fanning and vice versa), and increased rates of egg cannibalism (Behrens, Maciejewski, et al. 2025; Behrens, Tucker, et al. 2025).

While our understanding of this system is growing, it remains unclear whether white and common stickleback represent distinct ecotypes or incipient species. White and common stickleback are fully interfertile in advanced generation laboratory crosses (Behrens, Maciejewski, et al. 2025; Behrens, Tucker, et al. 2025; Blouw 1996), suggesting little intrinsic postzygotic isolation, although it is hard to be certain from existing evidence, and ecological postzygotic isolation has not been formally tested. An allozyme study found no evidence of genetic differentiation between the two types (Haglund et al. 1990). However, a distinct class of small‐bodied females is always found at sites with small‐bodied male white stickleback (Blouw and Hagen 1990; Jamieson et al. 1992a). Mate‐choice experiments and field observations suggest that these small females and males mate assortatively (Jamieson et al. 1992b, 1992a). This is consistent with experimental evidence of the role of body size in mate choice in other threespine stickleback (Conte and Schluter 2013), and suggests there may be premating barriers to gene flow in this system.

These findings suggest that white stickleback may be a fruitful system for studying recent and ongoing speciation. However, assessing the stage of speciation (recent or old), as well as the presence of ongoing gene flow, requires a detailed study of the genetic relationship between the white and common stickleback using modern tools. Here, we employ genetic, morphological, and isotopic data to explore the evolutionary history of the white stickleback. We adopt two a priori criteria for evaluating whether white and common stickleback represent distinct lineages with incomplete reproductive isolation: (1) genome‐wide genetic differentiation distributed across multiple chromosomes rather than at a single locus, and (2) the absence of detectable early‐generation hybrid individuals despite broad sympatry. We sought to answer the following two main questions:

First, do white stickleback represent a nascent species or a complex intrapopulation polymorphism? To answer this question, we examined patterns of genetic polymorphism in wild‐caught samples of white and common stickleback. If white and common stickleback represent separate, distinct lineages in broad sympatry, we expect divergence at sites across the genome rather than at a single site such as is seen in social and reproductive polymorphisms (e.g., Küpper et al. 2016).

Second, have white and common stickleback diverged along a trophic ecological axis, as in the limnetic‐benthic species pairs? To answer this question, we examined morphological traits and stable isotopes known to be associated with differences in diet and habitat in other threespine stickleback. If white and common stickleback have, like other stickleback species pairs, diverged along a trophic axis, we would expect them to display significant differences in ecomorphological traits and isotopic ratios.

By answering these questions, we hope to better understand the early stages of speciation in stickleback and lay the groundwork for developing the white stickleback into a full‐fledged study system.

## 2 Methods

### 2.1 Sample Collection

**FIGURE 1:**
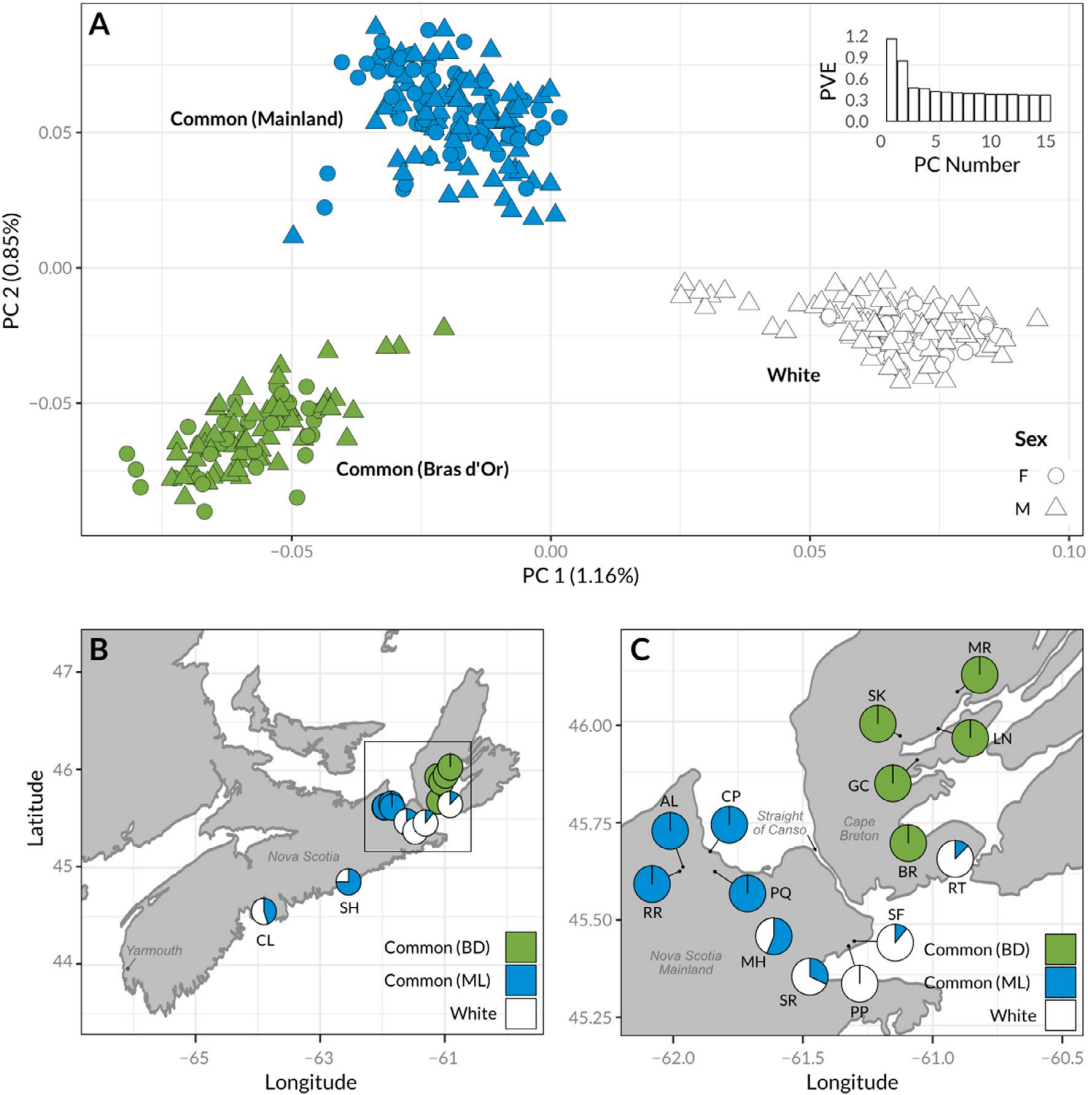
Male and female white stickleback form a distinct genotypic cluster. (A) Principal component analysis of ~8000 LD‐pruned SNPs derived from GBS reads. Colors indicate K‐means cluster groupings (*K* = 3). (B, C) Maps of the geographic distribution of genotypic clusters in Nova Scotia, Canada. Pie chart sections represent the proportion individuals at each site belonging to each genotypic cluster in A. Site labels: AL = Antigonish Landing, BR = Black River, CL = Canal Lake, CP = Captain's Pond, GC = Gillies Cove, LN = Little Narrows, MH = Milford Haven River, MR = Middle River, PP = Porper Pond, PQ = Pomquet, RR = Rights River, RT = River Tillard, SF = St. Francis Harbor, SH = Sheet Harbour, SK = Skye River, SR = Salmon River.

In early May–July of 2012 and 2014, we collected white and common threespine stickleback at 16 sites in Nova Scotia, Canada (Table S1). Locations included 11 sites adjacent to the mainland of Nova Scotia and five sites within Bras d'Or Lake, an inland sea on Cape Breton Island. We determined sites using the list of sites in Jamieson et al. (1992a) as a guide. We focused on sites where both types were most likely to co‐occur during the breeding season according to Blouw's environmental analysis: brackish water with abundant filamentous algae. This sampling scheme ultimately resulted in examining every accessible freshwater estuary we could access by car or short hike along the southern coast of Nova Scotia, from Yarmouth and through the Strait of Canso to Antigonish. In Bras d'Or Lake (2014 only), we sampled estuarine sites (where rivers or other freshwater bodies mixed into the sea) that we could access within a radius of approximately 100 km centered on the town of Whycocomagh, NS (Figure 1C).

At all sites, we caught fish by setting unbaited “Gee” brand ¼ inch mesh stainless steel minnow traps in shallow regions where we observed males courting females. These were set according to the general methods described in Schluter and McPhail (1992). Upon retrieving the traps, we evenly sampled phenotypically white and common males (identified by breeding color) and kept all females (identified by gravidity) until we had approximately 16 of each type of male and 32 unclassified females from each site. If we could not sex an individual by color or gravidity, or if a male had faded breeding colors, we did not collect it. All fish were euthanized using 0.5 g/L tricaine methanesulfonate (MS‐222) in seawater.

We placed all the individuals from each site into a single 1 L Nalgene container containing non‐denatured 95% ethanol and moved each fish to an individual 50 mL Falcon tube containing 95% ethanol a maximum of 6 h later. Upon returning from the field (4–6 weeks after collections), we removed the pectoral and tail fins of each individual and placed the clips in 1.5 mL microcentrifuge tubes filled with 95% ethanol.

### 2.2 Genotyping

We extracted DNA from the clipped fins of each individual using the protocol described in Peichel et al. (2001). Briefly, the tails were digested with proteinase‐K and we used a standard phenol‐chloroform extraction to isolate DNA. We eluted the resultant DNA in 1× TE and assessed DNA concentrations using a Qubit fluorometer (Qiagen Corp, Germany). After DNA quality control, we retained DNA from 365 individuals.

We then prepared three genotyping‐by‐sequencing (GBS) libraries using an adapted version of the original protocol (Elshire et al. 2011; Mondon et al. 2018). The first library contained DNA from 96 males from 2012, randomized in 96‐plate well position. Based on the number of high‐quality variants identified in the 2012 data, we increased the number of individuals to 148 for the second and third libraries. The two latter libraries contained DNA from a total of 296 males and females from 2014, randomized among library, plate and in 96‐plate well position. We aimed for an insert size of 300–400 base pairs and used a gel‐extraction method to size‐select fragments from the prepared libraries. We confirmed the final fragment size distribution using a Bioanalyzer (Agilent Technologies, California). The completed libraries were then sequenced in individual lanes of an Illumina HiSeq 2000 at the University of British Columbia Biodiversity Next Gen Sequencing facility.

### 2.3 Variant Identification

We identified variants using a pipeline adapted from the GATK 3.3.0 best practices guidelines (DePristo et al. 2011; McKenna et al. 2010). After demultiplexing the data using a Perl script, we used Trimmomatic version 0.32 (Bolger et al. 2014) to trim and filter sequences for quality. We then aligned the filtered reads to the stickleback reference genome v3 (Glazer et al. 2015) using BWA version 0.7.10 “mem” algorithm (Li and Durbin 2010). We then realigned these reads using the GATK version 3.3.0 RealignTargetCreator, and IndelRealigner. Finally, we identified variants using the HaplotypeCaller and genotyped the entire dataset using GenotypeGVCFs. To facilitate analyses that required an outgroup (e.g., TREEMIX) we also identified variants from whole genome data from six marine individuals from Denmark (Ferchaud et al. 2014) and a collection of populations from the west coast (Catchen et al. 2013; Samuk et al. 2017). We processed these using the same pipeline, but with a separate run of GenotypeGVCFs.

We combined the final VCFs from the Nova Scotia and outgroup samples using the “merge” function in bcftools (Li et al. 2009). To simplify later analyses, we only included sites with biallelic single nucleotide polymorphisms (SNPs). For all analyses, we also required sites to have genotype calls in at least 80% of all individuals.

We filtered this final dataset using slightly different criteria for each analysis (described below). These filters were meant to reduce bias and/or facilitate specific statistical analyses (e.g., by reducing interdependence in the data). Unless otherwise stated, we excluded SNPs with a minor allele frequency (MAF) of < 0.05 (after Linck and Battey 2019). For analysis requiring statistically independent sites, we pruned the dataset to reduce linkage disequilibrium (LD) between sites. This was done using the *snpgdsLDpruning*() function in the R package *SNPRelate* (Zheng 2012). This function calculates pairwise linkage disequilibrium (*r* ^2^) between all SNPs in a window of 5 SNPs, which is incremented along the genome at 5 SNP increments. If any SNPs in the window exceed the LD threshold (0.2), a single SNP is randomly chosen to be representative of SNPs in that window and the others are dropped. This reduces the statistical interdependence between SNPs caused by physical linkage, which is undesirable for most types of phylogenetic and demographic inference (Pickrell and Pritchard 2012). The final dataset ranged from 19,000–55,000 high‐quality SNPs in 354 individuals, depending on the filtration applied and the populations included.

Threespine stickleback have chromosomal sex determination, with males as the heterogametic sex (coded as XY, as in humans). The male sex chromosome also shares a small pseudoautosomal region with the X chromosome (White et al. 2015). Because of our reduced representation approach and poor sequencing of the Y, we chose to exclude the Y chromosome from our analysis. A more detailed analysis of the Y‐chromosome of these populations using whole genome data will be presented in a forthcoming paper (Sumarli et al. 2025).

### 2.4 Genetic Clustering: PCA, fastSTRUCTURE, and TREEMIX

To assess whether white stickleback represent a distinct genotypic cluster, we first ordinated the pruned SNP data using principal components analysis (PCA) using the R package *snpRelate* (Zheng 2012). We also examined the fit of fastSTRUCTURE models to the genetic data using the approach described in Raj et al. (2014). To further complement the clustering analyses and assess the presence of gene flow, we additionally performed tree and migration edge estimating using TREEMIX (Pickrell and Pritchard 2012). We performed this analysis using a pruned dataset, only including individuals collected in 2014, as well as outgroup samples from Denmark and British Columbia (from Samuk et al. 2017). To assess confidence in the topology of this tree, we performed 1000 bootstrap replicates of the tree fit using the included bootstrapping mode in TREEMIX. We then combined the bootstrap trees into a consensus tree.

To test for the signal of admixture, we estimated a base tree using the Denmark samples as an outgroup, followed by sequential addition of migration edges (starting with 0). For each new migration edge, we compared the goodness of fit of the new tree to the previous tree using a likelihood ratio test (Pickrell and Pritchard 2012). We classified the “best” tree as the last tree to offer a significant increase in likelihood. In terms of general patterns, the PCA and fastSTRUCTURE results were all highly concordant (Figure 1, Figure S2), and we thus chose to focus on the PCA results for brevity.

### 2.5 Localizing the Signatures of Divergence

In the presence of gene flow, loci involved in reproductive isolation between two populations are predicted to exhibit unusually elevated divergence, for example, higher F_ST_. Further, if a single large region or single locus is responsible for divergence between two populations, these extreme values should be highly localized in the genome—for example clustered in an inverted region (e.g., Küpper et al. 2016).

In order to localize the genomic regions involved in divergence between white and common stickleback, we performed an F_ST_ outlier analysis using the R package OUTFLANK (Whitlock and Lotterhos 2015). This method uses a parametric bootstrap to infer the expected neutral distribution of F_ST_ (inferred via fitting a chi‐squared distribution to a pruned set of SNPs) and compares the observed values of F_ST_ to this expectation to determine outlier status on a per‐SNP basis. We also separately performed this analysis for the X chromosome (chromosome XIX) using SNPs from only female individuals. We did this to account for differences in (a) effective population size and (b) male–female coverage on chromosome XIX.

### 2.6 Body Shape

To measure body shape, we determined the coordinates of morphometric landmarks on digital photographs of freshly euthanized individual fish (taken on the day of collection, prior to ethanol fixation) using *imageJ* (Rasband 2012). We used the landmarks described in Sharpe et al. (2008); see Figure 4B. We then imported the coordinates of these landmarks into *R* where we analyzed them using the *geomorph* 2.0 package (Sherratt et al. 2014). We performed generalized Procrustes analysis to align and scale the landmarks, followed by principal components analysis to identify the major axes of variation. We examined the first six principal components, which accounted for the majority (77%) of the variation in body shape. As found in other studies (Albert et al. 2008), the first principal component (PC1) represented differences in the degree of bending in specimens due to preservation. We thus restricted our analysis to PCs 2–5.

### 2.7 Skeletal Traits

To measure skeletal traits, we first stained the fish using Alizarin red following the protocol in Arnegard et al. (2014). We then took digital photographs of the stained specimens, counted the number of lateral armor plates, and then measured the length of spines using *ImageJ* (Rasband 2012). To count gill rakers, we dissected out the first gill arch and examined it under a dissecting microscope. We then counted the number of short and long gill rakers, again following the methods of (Arnegard et al. 2014).

### 2.8 Body Brightness

We quantified the brightness of the body by cropping a 1 cm^2^ section of the flank of each fish from a digital photograph taken under constant lighting conditions using *ImageJ*. We then obtained the mean RGB values (0–255 for each channel) for these segments using *ImageJ* and calculated an overall luminance score: *R* + G + B/(255*3).

### 2.9 Testes and Egg Mass

We quantified testes mass by first dissecting testes from each preserved male and drying them for 36 h in a glass desiccator containing granular desiccant. We then weighed the dried testes using a XS3DU microbalance (Mettler‐Tolledo, Ohio). When both testes were developed, we weighed both and took the average of the resulting measurement; otherwise, we measured the single developed testis. We quantified egg size by extracting up to ten individual eggs from each female and weighing them in the same manner as the testes. We divided the final weight by the number of eggs measured to obtain an average egg weight for each female. We note that ethanol preservation leaches lipids from tissues, which could reduce overall testes and egg mass and potentially reduce within‐ecotype variance in these measures. However, because both ecotypes were preserved identically, any such lipid extraction would affect both equally and would not bias the direction of ecotype differences. Lipid content per se may also be a biologically meaningful axis of variation in egg quality between ecotypes, a question we leave to future work.

### 2.10 Statistical Analysis of Morphological Data

We compared each morphological trait between white and common stickleback using a linear model (analysis of covariance). Because many of the traits we measured are known to covary strongly with body size, we first examined the relationship between body size (as measured by standard length or the “centroid‐size” value from the Procrustes analysis, whichever was more complete) and each trait by fitting a standardized major axis regression (i.e., model II regression) using the R package *smatr* (Warton et al. 2012). If a trait displayed at least a moderately strong, significant relationship with body size (*R* ^2^ > 0.4, *p* < 0.05), all further analyses were carried out via standardized major axis regression with body size as a covariate using *smatr*. Otherwise, we used standard linear models with the independent trait variable modeled as Gaussian for continuous traits or Poisson for count‐based traits. When traits were measured in both sexes, we also included sex (M/F) as a covariate. For purposes of comparison between traits, we standardized all trait values by subtracting the mean and dividing by the standard deviation of the combined dataset using the R function *scale*().

An important caveat with this analysis is that the white and common stickleback are known to differ in body size, with whites being smaller than commons (Blouw and Hagen 1990). This makes it difficult to statistically control fully for body size, as in some samples the two ecotypes have limited overlap in body size. In these cases, regression estimates of trait differences between ecotypes will be inexorably influenced by differences in body size, even after statistical control. The combined major axis and standard regression approach above ameliorates this issue but cannot eliminate it. As such, controlling for body size in this way may result in an underestimate of size‐independent ecotype differences. One way to account for this in future studies would be to examine development of these traits across life stages for both whites and commons.

### 2.11 Stable Isotopes

We quantified Carbon‐13/Carbon‐12 and Nitrogen‐15/Nitrogen‐14 isotopic ratios for white and common following the general method described in (Reimchen et al. 2008). Isotopic ratios of both elements have been shown to correlate with trophic position and diet in coastal marine ecosystems (France 1995; Lerner et al. 2022). Carbon ratios can distinguish among the type of primary producer a herbivore consumes, whereas nitrogen ratios can be indicative of trophic level (France 1995; Lerner et al. 2022). We began by dissecting out the dorsal muscle from each fish, drying it in a desiccator as above, and weighing out exactly 1.00 mg of dried tissue from each individual using a microbalance. We placed each unit of weighed tissue into an individual nickel capsule. The capsules were placed in a 96‐well plate, and shipped to the UC Davis Stable Isotope Facility for Carbon‐13/Carbon‐12 and Nitrogen‐ 15/Nitrogen‐14 ratio quantification. We did not obtain environmental samples for calibration of the isotopic ratios, and instead focused on relative isotopic differences between the whites and commons. We compared differences in Carbon and Nitrogen ratios together using a MANOVA via the *manova*() function in R (R Core Team 2018). We included sampling location as a covariate to control for geographic variance in isotopic abundances.

## 3 Results

### 3.1 Genotypic Clustering

The first two axes of our principal components analysis of the genomic data revealed three distinct genotypic clusters in the data set (Figure 1A). Males in ones of these clusters (white dots in Figure 1A) were all individuals that we had classified phenotypically as “white” in the field (light breeding colors and caught in areas with filamentous algae) in both 2012 and 2014. Two of the genotypic clusters (i.e., white and common) co‐occurred at many sites across Nova Scotia (Figure 1B,C), suggesting they do not represent neutral geographic structure. We found no clear intermediates, suggesting that our sampling did not identify any early‐generation hybrids, although a formal visualization of hybrid status (e.g., a triangle plot) is not possible because of the extremely low level of genetic divergence and lack of diagnostic differences between whites and commons (see later F_ST_ outlier analysis).

A third cluster (green dots, Figure 1A) consisted of stickleback exclusively from the geographically separate Bras d'Or region (Figure 1C). This cluster also contained three males that we scored phenotypically as “white” in the field, suggesting that either our field scores of phenotype were incorrect or a white male phenotype exists at a low level in the Bras d'Or population. These three individuals scored as phenotypically white did not form a separate cluster in higher PC dimensions. Note that, since that initial observation, we and others working on the white stickleback have never observed white stickleback in the Bras d'Or Lake region (C. Behrens, A. Dalziel and F. Chain, pers. comm.).

The general results of the PCA were closely mirrored by fastSTRUCTURE (Figure S2), with the best K value being 3, determined by method of Evanno et al. (2005). Two of these three clusters again corresponded to samples with phenotypically white or common fish (males and females) from the mainland. The third distinct cluster contained all individuals sampled in the Brad d'Or Lake. At *K* = 3, most individuals in all clusters had low levels of mixed ancestry, although there were again no obvious F1 or backcross hybrid individuals with *q* ≈ 0.5 or *q* ≈ 0.25/0.75 for any ancestry source (Figure S2, K = 3).

**FIGURE 2:**
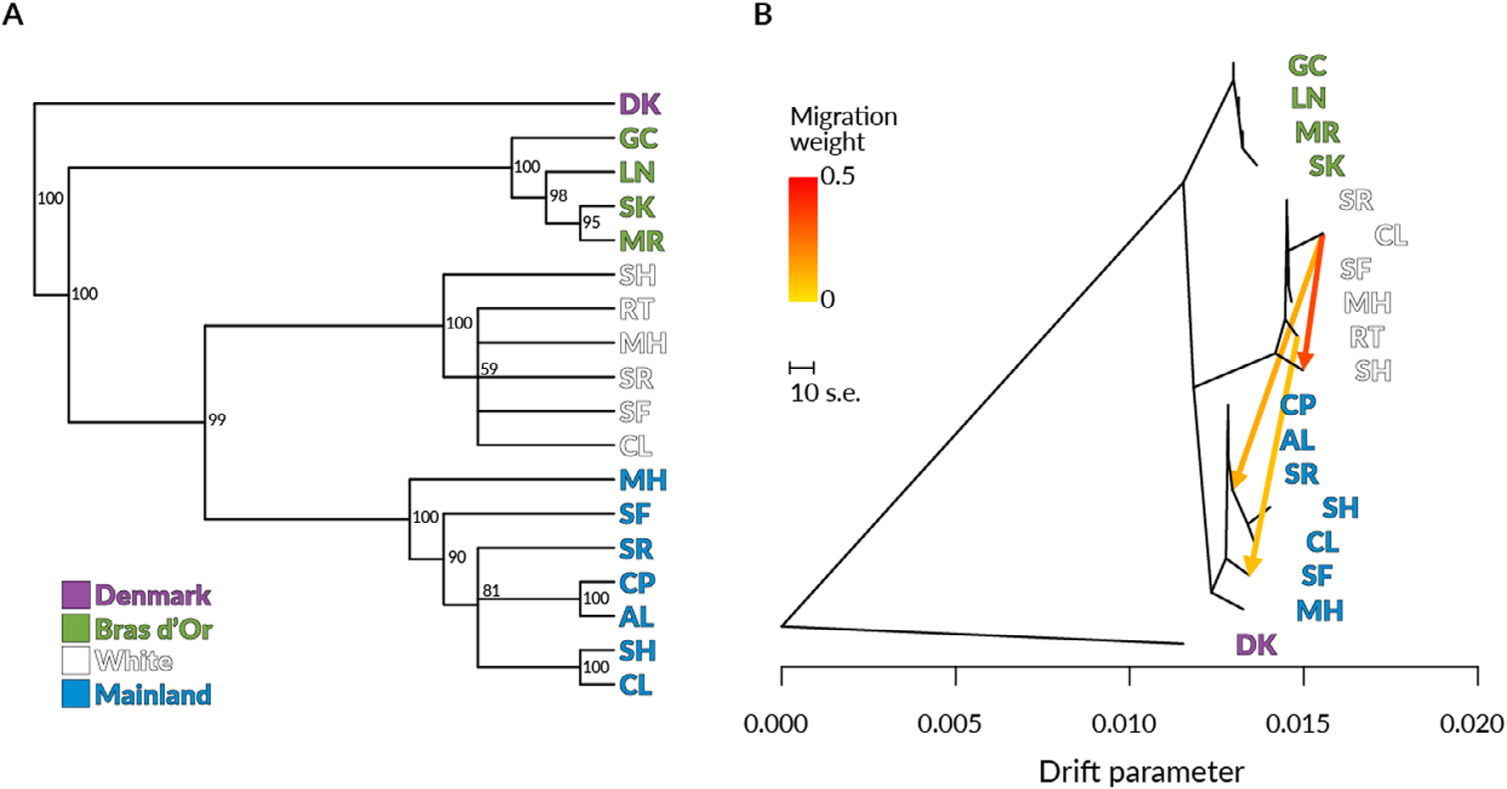
TREEMIX trees for Nova Scotian stickleback sampled in 2014, with a Denmark marine population as the outgroup. (A) A consensus tree derived from 1000 bootstrap replicates, assuming no migration. Node labels represent the percentage of trees in which each node exists. Larger numbers represent more confidence in the node. Branch lengths are arbitrary. Tip label colors correspond to genotypic clusters in Figure 1. (B) The maximum likelihood TREEMIX (m = 3 migration edges) tree. Migration edges are colored according to their weight (more red = higher relative migration). The drift parameter corresponds to the estimated amount of genetic drift that has occurred between populations.

Like PCA and fastSTRUCTURE, the consensus TREEMIX tree recovered three groups of Nova Scotian stickleback (Figure 2). The white, common and Bras d'Or clades all have strong support for their individual monophyly and strong support for whites and mainland commons as relatively young sister groups (Figure 2A, 0.85–1.00 bootstrap support). The best fit for number of migration edges for the Nova Scotia/Denmark tree was three (Figure 2A, likelihood ratio test: *χ* ^2^_1_ = 15.52, *p* = 0.000081). The strongest migration edge connected a western white population (Canal Lake) to another white population to the east (Sheet Harbour) (Figure 2B, red edge). The remaining two edges connected the white clade to Guysborough common populations, mirroring the signal of potential admixture from the fastSTRUCTURE analysis (Figure 2B, yellow edges).

### 3.2 F_ST_ Outlier Analysis

**FIGURE 3:**
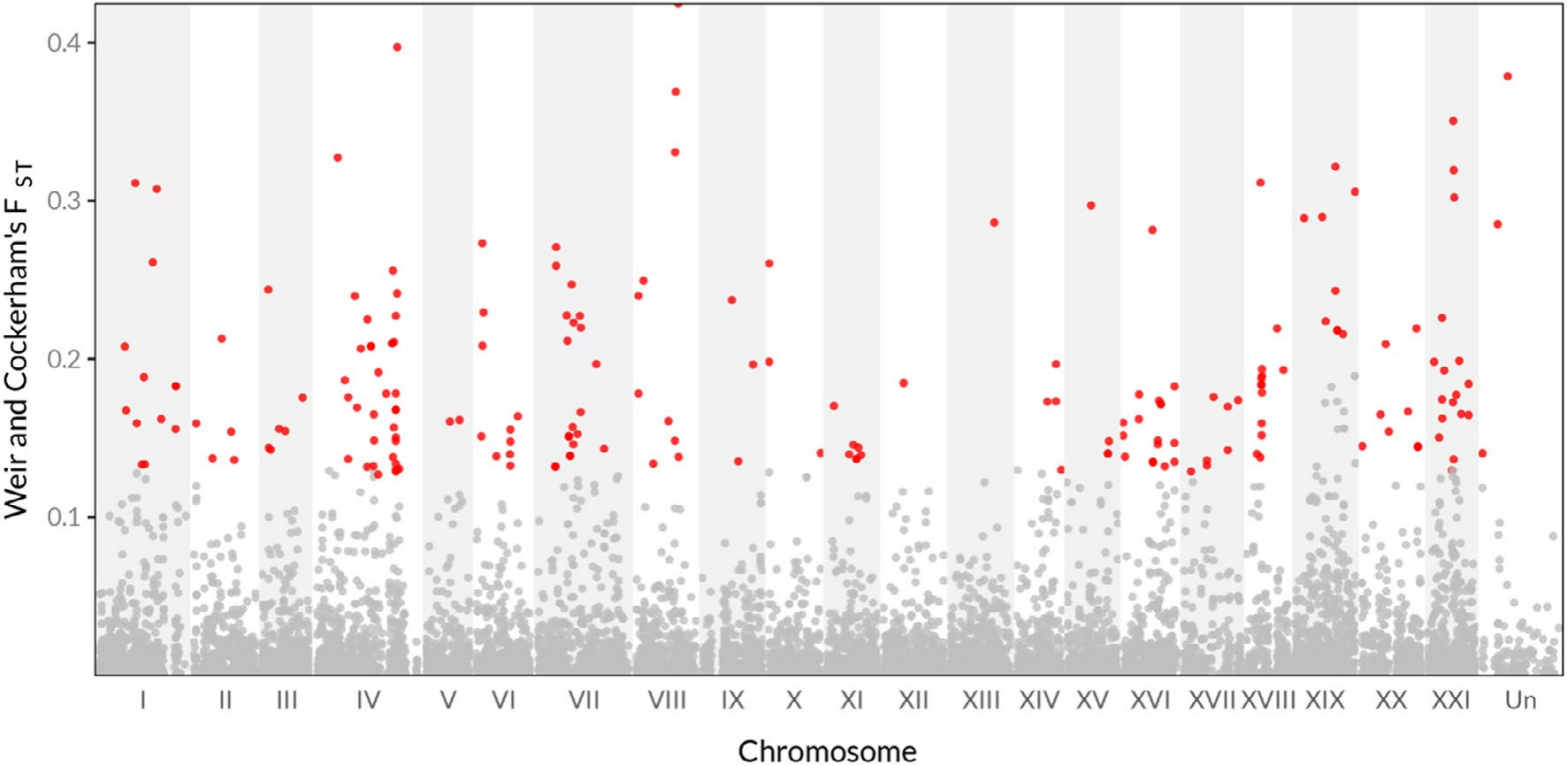
Weir and Cockerham's F_ST_ values across the genome for the comparison of mainland white and common stickleback. Chromosomes in the reference genome (roman numerals, alternatively shaded), are plotted in genomic order with “Un” representing concatenated unplaced scaffolds (Glazer et al. 2015 assembly). Gray points represent non‐outlier SNPs, and red points represent outlier SNPs identified via OUTFLANK.

Because we were primarily interested in the divergence between whites and commons per se, and not the effect of the geographic barriers to the Bras d'Or lake, we elected to restrict the F_ST_ analysis to whites and commons from the mainland only. Genetic divergence between these white and common stickleback occurred at many loci across the genome (Figure 3). Genome wide, we estimated Weir and Cockerham's F_ST_ between these groups to be approximately 0.01 (95% confidence interval 0.0102–0.0110). In absolute terms, this indicates very little divergence between white and common stickleback: compare with the value of 0.4 between sympatric benthic and limnetic species pairs (Taylor and McPhail 2000; Samuk et al. 2017). Several outlier loci displayed unusually high F_ST_, with one exceeding 0.4 (Figure 3, chromosome VIII). Interestingly, these F_ST_ outliers were widespread and found at many loci across every chromosome (Figure 3). There was also no indication of the action of a single, linked block (e.g., an inversion) underlying the pattern of divergence between white and common stickleback. Divergence between the two types appears to be a genome‐wide phenomenon, suggesting that the white and common stickleback are not simply morphs of a single polymorphic population.

### 3.3 Morphological Traits

**FIGURE 4:**
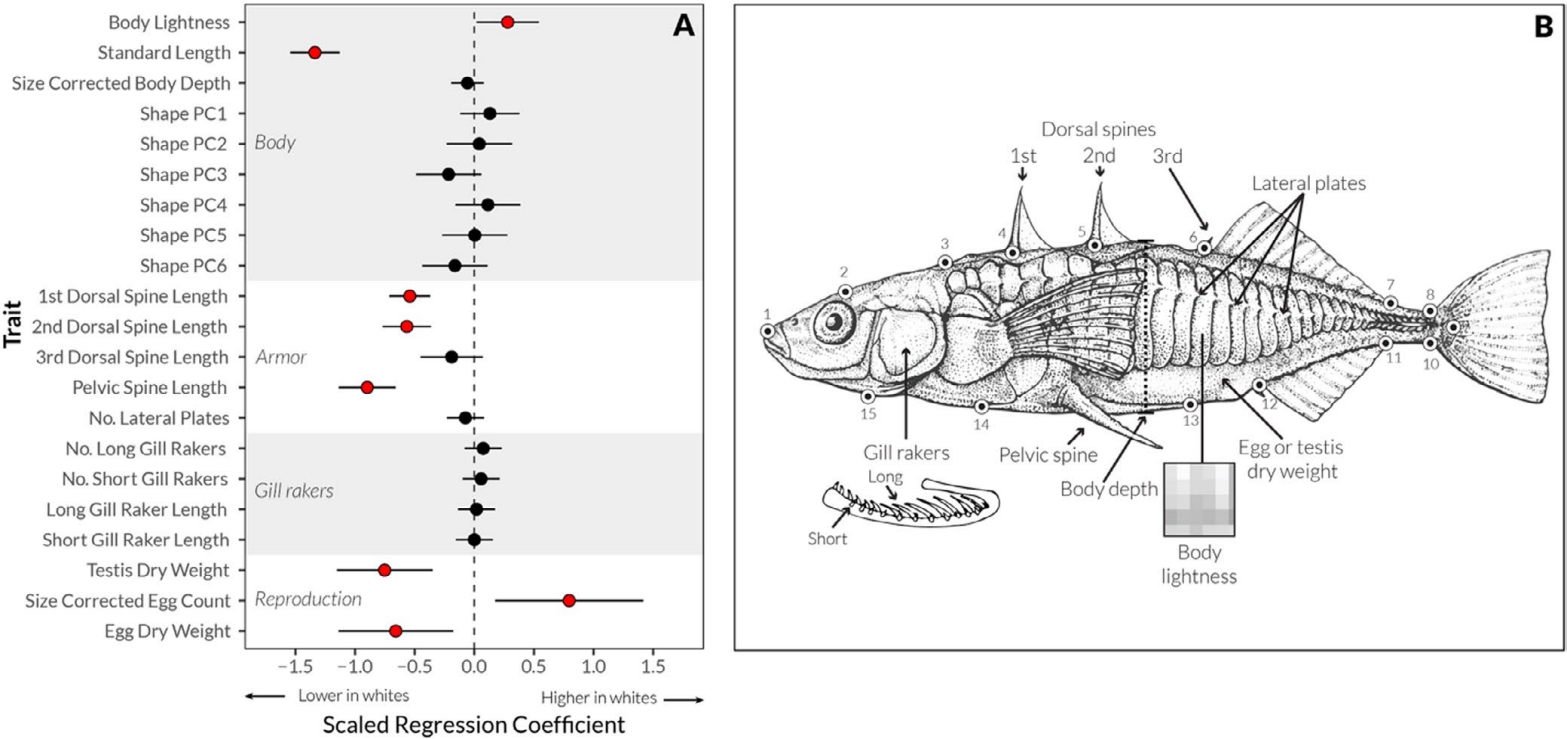
Morphological differences between white and common stickleback across a collection of traits with ecological and reproductive importance. (A) Model II regression coefficients of linear models fits (*x*‐axis, units are pooled standard deviations for across all individuals) and 95% confidence intervals for differences in trait values between white and common stickleback. Regression coefficients include correction for the effect of both body size and sex (where applicable). “Shape PC1‐6” traits correspond to scores on the first six size‐corrected principal components of the morphometric landmarks shown in (B) and displayed in Figure S1. (B) A schematic of the morphological traits measured as part of the study. Artwork modified from Foster and Bell (1994) with permission.

White and common stickleback did not differ in most of the morphological characters classically associated with ecological differences in threespine stickleback, including body shape, armor plates, and gill rakers (Figure 4A). We note that these “classic” ecological associations were largely established in freshwater lake populations; it remains possible that different traits are ecologically relevant in the marine environment studied here. White and common stickleback nevertheless displayed moderate to large differences in a number of other morphological characters (Figure 4A). Consistent with previous work, we found that white stickleback are on average approximately 1.5 standard deviations (~1.5 cm) smaller than common stickleback. Both male and female white stickleback also tend to be significantly brighter in overall color and have moderately smaller dorsal and pelvic spines than common stickleback, although this difference appeared to be highly sensitive to statistical control for body size (see methods). Finally, white and common stickleback exhibit a number of significant differences in reproduction‐associated traits: white stickleback have smaller testes and eggs, but a larger number of eggs than the common form (after accounting for body size, Figure 4A).

### 3.4 Stable Isotopes

**FIGURE 5:**
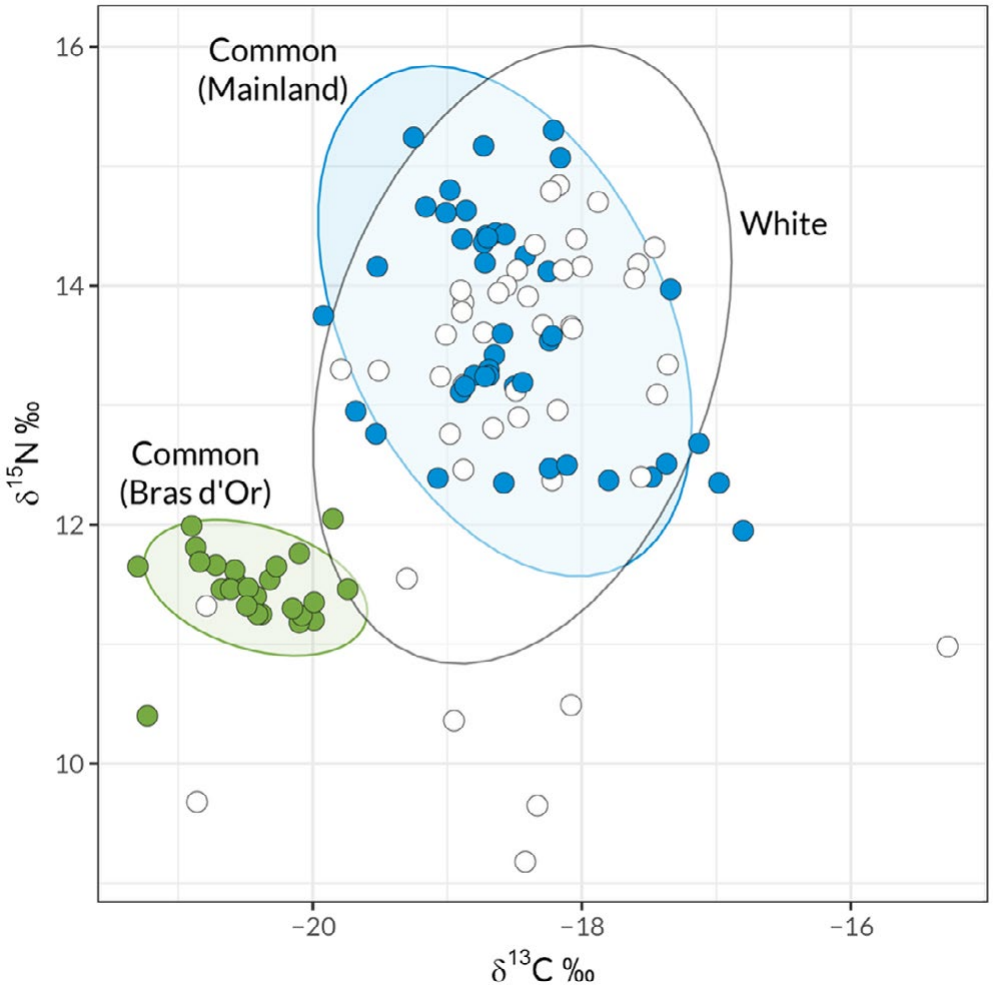
The relative abundance of δ ^13^C and δ ^15^N stable isotopes in the muscle tissue of stickleback collected throughout Nova Scotia. Each point represents the isotopic ratio in a single individual. Points are colored by genetic cluster as identified in Figure 1A. Shaded ellipses represent 95% confidence ellipses for reach group.

White and common stickleback do not differ in their Carbon and Nitrogen isotopic ratio profiles (Figure 5). Samples from both ecotypes show broadly overlapping distributions of both C and N ratios, and do not differ significantly in multivariate distribution (MANOVA, species term: Pillai's Trace = 0.048, Approximate F_2,106_ = 2.0, *p* = 0.088). In contrast, there was a strong effect of sampling location on C and N ratios, with the Bras d'Or populations showing distinctly lower δ ^13^C and δ ^15^N ratios (Figure 5, MANOVA location term: Pillai's Trace = 1.18, Approximate F_8,214_ = 39, *p* < 2 × 10^−16^).

## 4 Discussion

Incipient species in the early stages of genetic divergence provide a unique window into the causes of speciation. However, identifying such systems is inherently difficult. Here, we used modern population genomics methods and attempted to clarify whether the “white” threespine stickleback of Nova Scotia—a phenotypically distinctive form known for over 40 years—represents a genetically distinct lineage. We found compelling evidence that the white stickleback is genetically distinct, but very closely related, to the common Atlantic marine stickleback ecotype. A low level of genome‐wide genetic divergence might be the result of ongoing gene flow and/or very recent divergence between the two types. We also found that white and common stickleback do not differ in most of the morphological characters classically associated with trophic divergence in stickleback except body size. The two forms were similar in body shape and gill rakers, in contrast to the sympatric limnetic and benthic species (Hatfield 1997). Their ecological similarity is reflected in their broadly overlapping stable isotope ratios. Instead, white stickleback appear to differ principally in male size, color and reproduction‐associated traits, as well as defensive spines. As such, unlike the sympatric stickleback species pairs and all other parapatric forms such as marine‐stream and lake‐stream stickleback pairs (Lavin and Mcphail 1993; Berner et al. 2009), the white stickleback appears to have diverged along alternative, potentially non‐ecological trait axes. We note that an absence of detected ecological differences does not rule out ecological divergence that we did not measure; there may be many other ecologically relevant axes (e.g., migration strategy, microhabitat use, thermal tolerance) along which whites and commons differ. A simpler alternative explanation is that the two ecotypes diverged allopatrically, accumulating differences through mutation‐order effects rather than through divergent selection, and have only recently come into sympatry. Instead, the white form's most distinctive attribute is the loss of paternal care and related reproductive differences. Recent experimental work has provided direct evidence that parental care differences act as a novel reproductive isolating mechanism in this system (Behrens, Maciejewski, et al. 2025). For this reason, sexual selection and/or ecologically mediated selection on reproductive traits may be important mechanisms driving divergence in these populations. Speciation driven by these mechanisms in the absence of conspicuous ecological/trophic differences is, as far as we know, without parallel in threespine stickleback. We note, however, an important caveat regarding causation: the observed differences in reproductive behavior could equally be a consequence of secondary contact rather than a cause of initial divergence. For example, differences in parental care could have evolved after contact as a form of reinforcement, or in direct response to reproductive interference (e.g., egg cannibalism between ecotypes; Behrens, Maciejewski, et al. 2025). Disentangling these alternatives will require careful demographic and experimental work.

### 4.1 White Stickleback as a Distinct Ecotype

While the population genetic methods we used indicate white stickleback are genotypically distinct from common stickleback, they are still very closely related to the sympatric common stickleback. The overall F_ST_ between white and common stickleback in Nova Scotia is only ~0.01. For a phenotypically distinguishable ecotype, this is unusually low compared to ~0.4 between sympatric benthics and limnetics (Taylor and McPhail 2000). It is much lower than the F_ST_ ≈ 0.2 seen between lake and stream ecotypes in British Columbia, which have relatively low levels of reproductive isolation and have not yet attained full sympatry (Roesti et al. 2012). We note that F_ST_ is a relative measure that scales with within‐population diversity; comparisons of F_ST_ values across systems with different effective population sizes or mutation rates should therefore be interpreted with caution.

According to the results of the TREEMIX analysis this high genetic similarity appears to be the result of very recent divergence—most likely much more recent than the split between the Eastern and Western Atlantic 17–37 kya (Fang et al. 2018). We note that the Fang et al. (2018) age estimate for the Atlantic‐Pacific stickleback split has been widely questioned, and Fang et al. (2020) themselves acknowledged it may be an underestimate. This divergence time estimate is also likely influenced by the apparent ongoing gene flow between whites and commons, and higher‐resolution genomic data will be needed to resolve their full demographic history.

In light of the recent divergence and evidence of gene flow, it is surprising that there do not appear to be any clear early‐generation hybrid individuals in our sample. For example, assuming that the genetic differences between white and common stickleback are numerous and found throughout the genome, hybrid individuals ought to have manifested as intermediates in the PCA projection, or as having large amounts (~50% for an F1) mixed ancestry in the fastSTRUCTURE plots. There are several possible explanations for a lack of hybrids. First, we did not collect ambiguous‐looking males, which may have been more likely to be hybrid individuals. This does not, however, account for a lack of hybrid females. It is also possible that our sampling method was somehow biased against finding hybrids. For example, perhaps hybrids have transgressive preferences for nest sites or timing of mating (such as they are in other reproductive traits, Behrens, Tucker, et al. 2025). Finally, it is possible that reproductive isolation between white and common stickleback, while recently evolved, has become nearly complete. Indeed, Jamieson's (1992a) laboratory and field trials suggested that there is near‐perfect assortative mating within white and common stickleback. If premating isolation is indeed as strong as these experiments suggest, it is perhaps not surprising that we did not detect any early‐generation hybrids. We also note that individuals at the periphery of the white and common clusters in Figure 1A could represent later‐generation backcross hybrids rather than pure‐type individuals (cf. Dean et al. 2024); and that even with gene flow, strong divergent selection can efficiently remove first‐generation hybrids from sympatric populations (Schluter et al. 2025).

A key open question concerns the biogeographic scenario by which the present sympatric situation arose. The striking geographic distribution of white stickleback—restricted to the south coast of Nova Scotia and co‐occurring with common stickleback there, while common stickleback occur alone on the north side—is consistent with more than one history. One plausible scenario is secondary contact following allopatric divergence: common stickleback may have spread through the Strait of Canso (likely dry as recently as ~6000 years ago; Vacchi et al. 2018) and come into secondary contact with a Bay of Fundy “white” population that had diverged in isolation (cf. Kitano et al. 2007, 2009). Alternatively, the two ecotypes may have diverged with ongoing gene flow in their current sympatric range. Demographic modeling in a companion whole‐genome study (Sumarli et al. 2025) explicitly compared these scenarios and found that a model of divergence with continuous gene flow provided a better fit to the data than a secondary contact model, providing support for the latter interpretation. We nonetheless consider both scenarios live possibilities, as demographic models have limited power to distinguish recent secondary contact from long‐standing divergence with gene flow, and additional work is needed to fully resolve this question.

### 4.2 Reproductive Strategy Polymorphism?

The hypothesis we address here is that the white stickleback represents a separate species from the common Atlantic threespine stickleback (Blouw and Hagen 1990). A key alternative explanation for the existence of a divergent white form of stickleback is that the two forms represent a male reproductive strategy polymorphism (Gross 1996; Taborsky et al. 2008; Mank 2023). There are three types of male alternative reproductive strategy that the white stickleback could represent: genetic, ontogenetic, and condition dependent. We address the evidence for each of these below.

The white stickleback is unlikely to represent a genetically determined alternative male strategy. Alternative male strategies are predicted to have a simple genetic basis, to preclude their breakdown by recombination (Gross 1996; Kopp and Hermisson 2006; Taborsky et al. 2008). Empirical evidence supports this prediction, with all known cases of complex genetic mating strategy polymorphisms being associated with a large non‐recombining region (Kopp and Hermisson 2006; Küpper et al. 2016; Purcell and Brelsford 2025). The genome‐wide differentiation between white and common stickleback is not consistent with this genetic architecture. Instead, white and common stickleback show genetic changes distributed over many loci and an overall reduction in gene flow despite sympatry—as expected in the case of incomplete and/or extremely recent reproductive isolation.

In contrast, a purely conditional strategy (e.g., low body condition triggers a switch in strategy) involving no genetic polymorphism would, by definition, not be strongly associated with genetic differences (Gross 1996). However, we observed that the sympatric pair of white and common stickleback is genetically differentiated on multiple chromosomes in both sexes at every geographic location sampled, arguing strongly against this possibility.

Finally, white stickleback are likely not an ontogenetically determined strategy (e.g., year one vs. year two breeding males), again because the polymorphism would not be strongly associated with genetic differences. Further, the genetic differences we see between white and common stickleback genetic clusters are stable through time (e.g., the “white” PCA cluster contains both 2012 and 2014 white stickleback), ruling out a cohort effect as the cause. Thus, our results are consistent with Blouw and Hagen's (1990) original hypothesis that white and common stickleback represent partially (or recently completely) reproductively isolated lineages rather than alternative male strategies.

### 4.3 Genetic Structure Within Nova Scotia

Our analyses revealed that common stickleback from the Bras d'Or Lake, an inland sea, are distinct from those on the outer coast (“mainland”). This is consistent with a growing appreciation for the phenotypic and genetic diversity of stickleback populations in Eastern Canada, which has been generally understudied (Haines 2023). Interestingly, in spite of previous findings by Blouw and colleagues who reported white stickleback in the Bras d'Or, none of the individuals sampled from Bras d'Or appeared to be genotypically similar to mainland white stickleback. This is made stranger by the fact that the handful of males we scored phenotypically as “white” at these populations failed to cluster with the rest of the white stickleback from the mainland. One possibility is that this is the result of the alleles that cause white nuptial colors segregating in the marine common Bras d'Or population. Perhaps persistent gene flow between Bras d'Or and whites (directly or via mainland commons) and/or balancing selection maintains a color polymorphism independent of the other loci and traits that characterize the white stickleback ecotype on the outer coast. Further work dissecting the genetics of the white/common difference and more extensive sampling in the Bras d'Or region will hopefully elucidate these issues.

### 4.4 The Potential of the White Stickleback Study System

Our findings suggest that the white stickleback is an excellent candidate system for studying the genetics of recent speciation. Indeed, work has already begun to dissect the genetic basis of some of the trait differences between whites and commons (Behrens, Maciejewski, et al. 2025). In addition, the white stickleback may serve as an excellent test‐bed for theories about the interacting roles of ecological and sexual selection in speciation. White stickleback appear to have diverged in a number of mating and/or reproductive‐related traits and not in most of the typical ecological traits. If this is indeed the case, sexual selection may have been an important driver of the evolution of reproductive isolation in this system. For example, white male coloration may have evolved as a sexual signal in concert with changes in nest site preferences for algae. While theory suggests that speciation via sexual selection alone is difficult in the face of gene flow (Servedio and Bürger 2014; Servedio and Kopp 2012), some models suggest that spatial variation in resources (e.g., nest sites) can dramatically increase the probability of speciation via sexual selection, with parental care being a potential target of selection (M'Gonigle et al. 2012; Reyes et al. 2025).

## 5 Conclusions

Here, we used genome‐wide genotyping, morphometrics and stable isotope analysis to explore whether white stickleback represent an ecologically‐differentiated, genetically distinct ecotype. We found that white stickleback are genetically distinct from common stickleback and that this involves multiple regions of the genome distributed over multiple chromosomes: despite low overall genomic divergence and evidence of gene flow, male and female white stickleback form a unique genotypic class, distinct from sympatric common stickleback. As such, white stickleback likely do not represent a genetically determined male mating strategy polymorphism, nor young or low‐condition males, but rather a distinct ecotype with as‐yet incomplete reproductive isolation (and potentially an incipient species). Finally, we found that white stickleback do not display all of the tell‐tale signs of ecological differentiation found in freshwater stickleback species pairs. Sexual selection (perhaps mediated by nest site preference) may be the key driver of reproductive isolation in this system.

## Author Contributions

**Kieran Samuk:** conceptualization (lead), data curation (lead), formal analysis (lead), funding acquisition (supporting), investigation (lead), methodology (lead), project administration (lead), resources (supporting), software (lead), visualization (lead), writing – original draft (lead), writing – review and editing (supporting). **Dolph Schluter:** conceptualization (equal), formal analysis (supporting), funding acquisition (lead), methodology (supporting), resources (lead), software (supporting), supervision (lead), writing – review and editing (equal). **Hannah Visty:** data curation (supporting), investigation (supporting), methodology (supporting), writing – review and editing (supporting).

## Funding

The work presented here was funded by a Canadian Natural Sciences and Engineering Research Council (NSERC) Discovery Grant to DS. KS was additionally funded by an NSERC Canada Graduate Scholarship.

## Conflicts of Interest

The authors declare no conflicts of interest.

## Supporting information


**Figure S1:** A scatterplot of a principal component scores of body shape from white (wht), common (cmn), and Cape Breton (Bras d'Or) stickleback. Dots are labeled according to their population genetic cluster assignments in Figure 1A. PCs 1–2 are shown in A, and PCs 3–4 are show in B. Thin plate spline warps and landmark positions are shown along each axis to show the range of shape variation (minimum and maximum) along each axis.
**Figure S2:** fastSTRUCTURE results for stickleback collected in Antigonish (AN), Cape Breton (Bras d'Or) (CB), Guysborough (GY), and Halifax (HA) regions, Nova Scotia, Canada in 2014. Each vertical bar within each subplot represents the ancestry proportions (q‐value) for a single individual. Ancestry proportions are colored to match clusters in Figure 1A (main text): blue = mainland common, green = Bras d'Or common, white = white stickleback. Additional colors were added for clusters of unknown origin (light blue and orange in *k* = 4 and *k* = 5) fastSTRUCTURE results are shown for *k* = 2 to *k* = 5 (rows).
**Table S1:** Decimal coordinates for collection sites for white and common stickleback in Nova Scotia, Canada. Full site names are given in the first column along with their corresponding codes used in Figure 1A. “Types Present” refers to whether we observed the presence of both white and common types of stickleback or only commons (there were no sites with only white stickleback).

## Data Availability

All code and data for the paper are available via the following Github repository: https://github.com/samuk‐lab/ws_ecology.
