# Supplementary material for: Genetic Divergence in the Absence of Strong Ecological Differences Between Coexisting White and Common Atlantic Marine Stickleback
Source: Ecol Evol. 2026 May 13;16(5):e73655. doi: 10.1002/ece3.73655 (PMC13171251; doi:10.1002/ece3.73655)
Supplement: Supplementary file 1 — Figure S1: A scatterplot of a principal component scores of body shape from white (wht), common (cmn), and Cape Breton (Bras d'Or) stickleback. Dots are labeled according to their population genetic cluster assignments in Figure 1A. PCs 1–2 are shown in A, and PCs 3–4 are show in B. Thin plate spline warps and landmark positions are shown along each axis to show the range of shape variation (minimum and maximum) along each axis. Figure S2: fastSTRUCTURE results for stickleback collected in Antigonish (AN), Cape Breton (Bras d'Or) (CB), Guysborough (GY), and Halifax (HA) regions, Nova Scotia, Canada in 2014. Each vertical bar within each subplot represents the ancestry proportions (q‐value) for a single individual. Ancestry proportions are colored to match clusters in Figure 1A (main text): blue = mainland common, green = Bras d'Or common, white = white stickleback. Additional colors were added for clusters of unknown origin (light blue and orange in k = 4 and k = 5) fastSTRUCTURE results are shown for k = 2 to k = 5 (rows). Table S1: Decimal coordinates for collection sites for white and common stickleback in Nova Scotia, Canada. Full site names are given in the first column along with their corresponding codes used in Figure 1A. “Types Present” refers to whether we observed the presence of both white and common types of stickleback or only commons (there were no sites with only white stickleback). [file ECE3-16-e73655-s001.docx]

## Supplemental Material


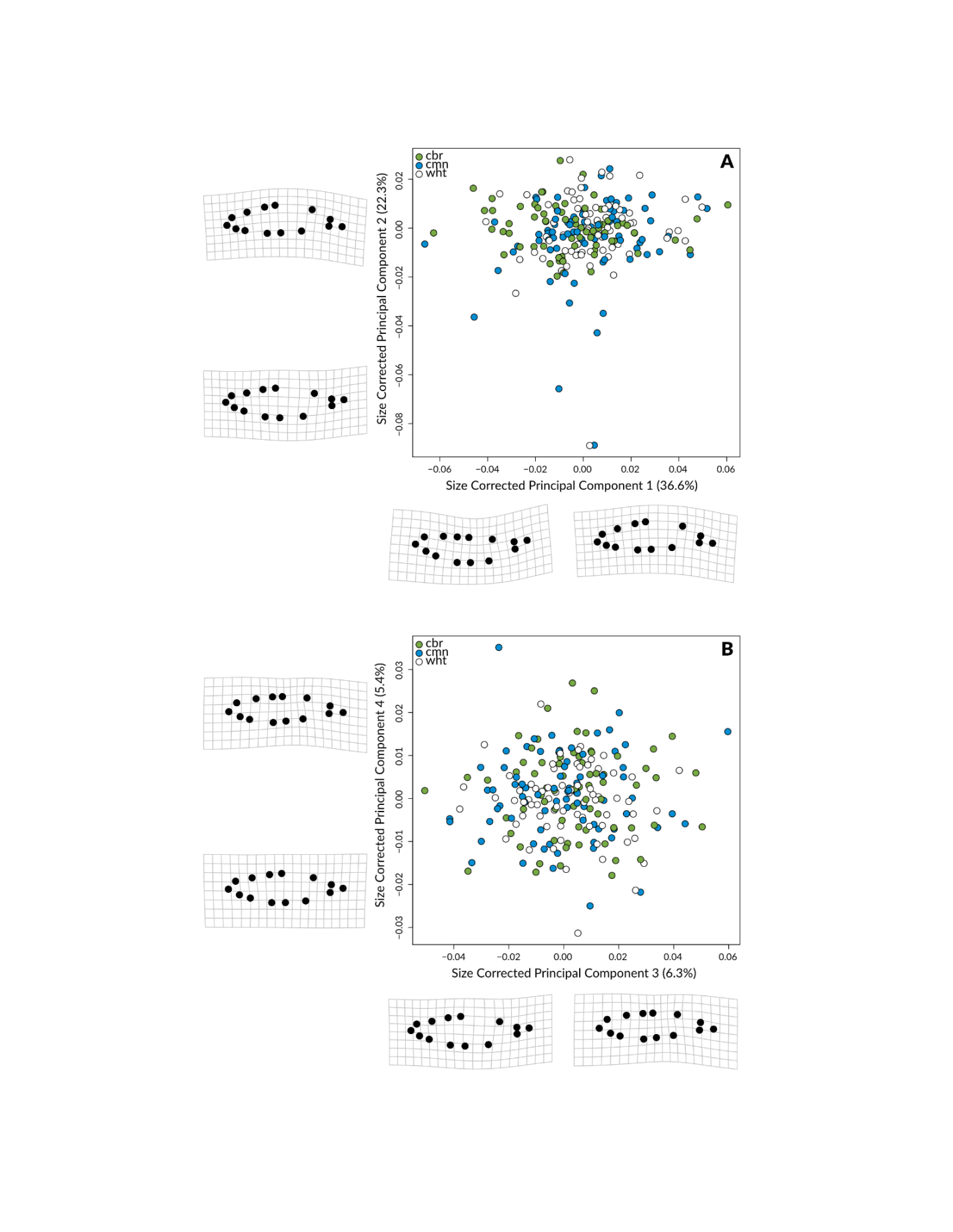


**Figure S1**| A scatterplot of a principal component scores of body shape from white (wht), common (cmn), and Cape Breton (Bras d’Or) stickleback. Dots are labelled according to their population genetic cluster assignments in Figure 1A. PCs 1-2 are shown in A, and PCs 3-4 are show in B. Thin plate spline warps and landmark positions are shown along each axis to show the range of shape variation (minimum and maximum) along each axis.


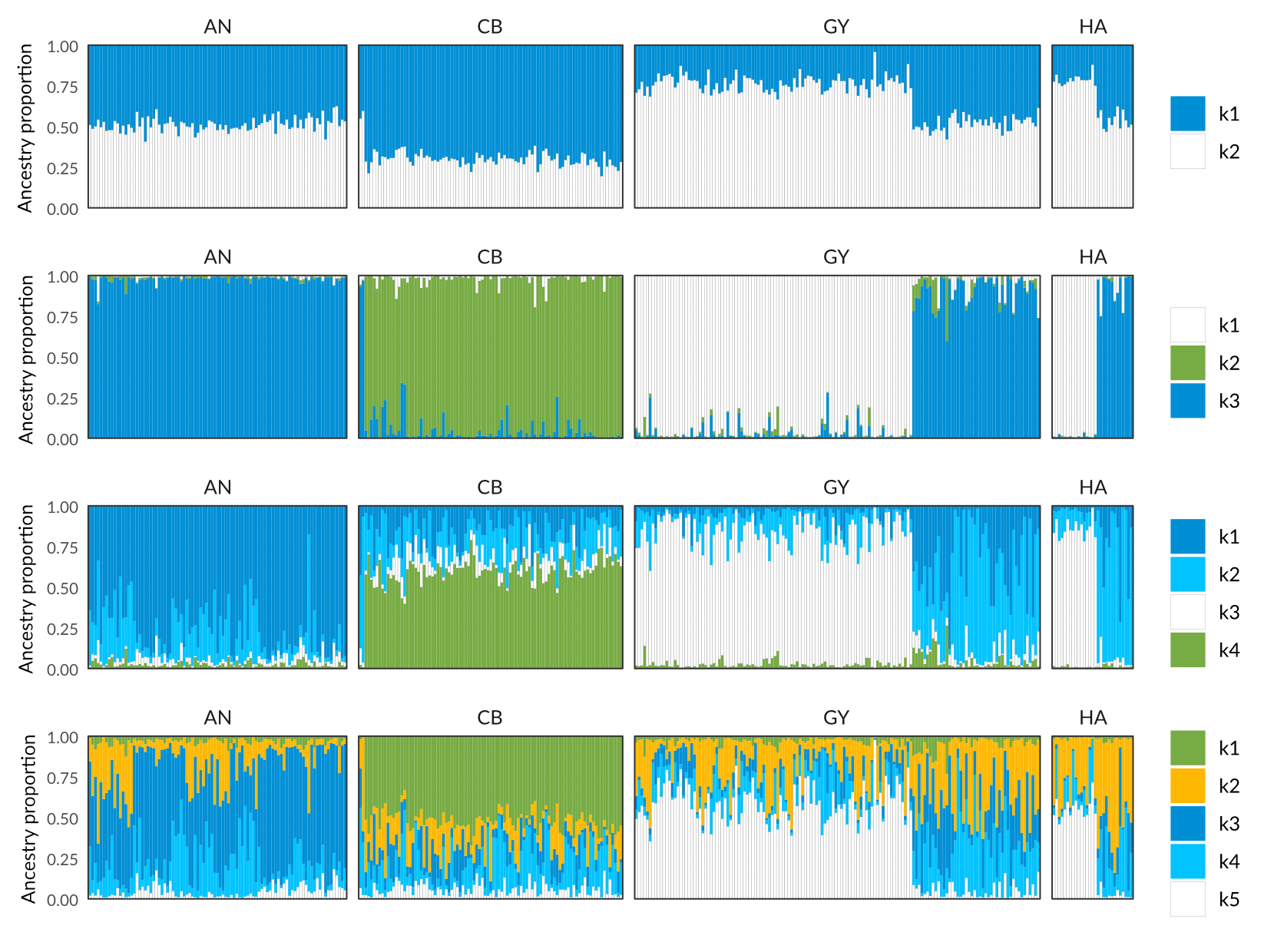
**Figure S2** | fastSTRUCTURE results for stickleback collected in Antigonish (AN), Cape Breton (Bras d’Or) (CB), Guysborough (GY), and Halifax (HA) regions, Nova Scotia, Canada in 2014. Each vertical bar within each subplot represents the ancestry proportions (q-value) for a single individual. Ancestry proportions are colored to match clusters in Figure 1A (main text): blue = mainland common, green = Bras d’Or common, white = white stickleback. Additional colors were added for clusters of unknown origin (light blue and orange in k=4 and k=5) fastSTRUCTURE results are shown for k=2 to k=5 (rows).

####

#### Table S1 | Decimal coordinates for collection sites for white and common stickleback in Nova Scotia, Canada. Full site names are given in the first column along with their corresponding codes used in Figure 1A. “Types Present” refers to whether we observed the presence of both white and common types of stickleback or only commons (there were no sites with only white stickleback).

| **Site Name** | **Types Present** | **Latitude** | **Longitude** |
| --- | --- | --- | --- |
| Porper's Pond (PP) | Both | 45.43719 | -61.326 |
| Captain's Pond (CP) | Common | 45.67189 | -61.8612 |
| Pomquet (PQ) | Common | 45.62684 | -61.8448 |
| Antigonish Landing (AL) | Common | 45.63197 | -61.96 |
| Rights River (RR) | Common | 45.62721 | -61.9688 |
| Canal Lake (CL) | Both | 44.49852 | -63.9034 |
| Milford Haven Collection (MH) | Both | 45.45772 | -61.6121 |
| West Sheet Harbour Pond (SH) | Both | 44.91992 | -62.5438 |
| Black River (BR) | Common | 45.69756 | -61.0938 |
| Gillis Cove (GC) | Both | 45.91452 | -61.0545 |
| River Tillard (RT) | Both | 45.65705 | -60.9131 |
| Skye River (SK) | Common | 45.97042 | -61.1193 |
| Little Narrows (LN) | Both | 45.99216 | -60.985 |
| Middle River West (MR) | Common | 46.08322 | -60.9103 |
| Salmon River Estuary (SR) | Both | 45.35266 | -61.4728 |
| St. Francis Harbour (SF) | Both | 45.44589 | -61.3089 |
